# Risk Factors for and Estimated Incidence of Community-associated *Clostridium difficile* Infection, North Carolina, USA^1^

**DOI:** 10.3201/eid1602.090953

**Published:** 2010-02

**Authors:** Preeta K. Kutty, Christopher W. Woods, Arlene C. Sena, Stephen R. Benoit, Susanna Naggie, Joyce Frederick, Sharon Evans, Jeffery Engel, L. Clifford McDonald

**Affiliations:** Centers for Disease Control and Prevention, Atlanta, Georgia, USA (P.K. Kutty, S.R. Benoit, L.C. McDonald); Department of Veterans Affairs Medical Center, Durham, North Carolina, USA (C.W. Woods, S. Naggie, J. Frederick); Duke University Medical Center, Durham (C.W. Woods, S. Naggie, S. Evans); Durham County Health Department, Durham (A.C. Sena); University of North Carolina, Chapel Hill, North Carolina, USA (A.C. Sena); North Carolina Department of Health and Human Services, Raleigh, North Carolina, USA (J. Engel); 1 Presented in part at the 44th Annual Meeting of the Infectious Diseases Society of America, Toronto, Ontario, Canada, October 12–15, 2006.

**Keywords:** Clostridium difficile, enteric infections, incidence, risk factors, community, bacteria, research

## Abstract

We determined estimated incidence of and risk factors for community-associated *Clostridium difficile* infection (CA-CDI) among patients treated at 6 North Carolina hospitals. CA-CDI case-patients were defined as adults (>18 years of age) with a positive stool test result for *C. difficile* toxin and no hospitalization within the prior 8 weeks. CA-CDI incidence was 21 and 46 per 100,000 person-years in Veterans Affairs (VA) outpatients and Durham County populations, respectively. VA case-patients were more likely than controls to have received antimicrobial drugs (adjusted odds ratio [aOR] 17.8, 95% confidence interval [CI] 6.6–48] and to have had a recent outpatient visit (aOR 5.1, 95% CI 1.5–17.9). County case-patients were more likely than controls to have received antimicrobial drugs (aOR 9.1, 95% CI 2.9–28.9), to have gastroesophageal reflux disease (aOR 11.2, 95% CI 1.9–64.2), and to have cardiac failure (aOR 3.8, 95% CI 1.1–13.7). Risk factors for CA-CDI overlap with those for healthcare-associated infection.

## Abstract 2

Antimicrobial drug exposure is the most common modifiable risk factor for infection.

## CME ACTIVITY

MedscapeCME is pleased to provide online continuing medical education (CME) for this journal article, allowing clinicians the opportunity to earn CME credit. This activity has been planned and implemented in accordance with the Essential Areas and policies of the Accreditation Council for Continuing Medical Education through the joint sponsorship of MedscapeCME and Emerging Infectious Diseases. MedscapeCME is accredited by the Accreditation Council for Continuing Medical Education (ACCME) to provide continuing medical education for physicians. MedscapeCME designates this educational activity for a maximum of 0.75 *AMA PRA Category 1 Credits*™. Physicians should only claim credit commensurate with the extent of their participation in the activity. All other clinicians completing this activity will be issued a certificate of participation. To participate in this journal CME activity: (1) review the learning objectives and author disclosures; (2) study the education content; (3) take the post-test and/or complete the evaluation at **http://www.medscape.com/cme/eid**; (4) view/print certificate.

## Learning Objectives

Upon completion of this activity, participants will be able to:

- Specify the prevalence of community-acquired *Clostridium difficile* infection
- Describe demographic trends in community-acquired *C. difficile* infection
- Identify case characteristics of *C. difficile* infection
- List risk factors for community-acquired *C. difficile* infection

## EDITOR

**Nancy Mannikko, PhD, MS, BS,** Copyeditor, Emerging Infectious Diseases. *Disclosure: Nancy Mannikko, PhD, MS, BS, has disclosed no relevant financial relationships.*

## CME AUTHOR

**Charles P. Vega, MD**, Associate Professor, Residency Director, Department of Family Medicine, University of California, Irvine, California, USA. *Disclosure: Charles P. Vega, MD, has disclosed no relevant financial relationships.*

## AUTHORS

Disclosures: **Preeta K. Kutty, MD, MPH; Arlene C. Sena, MD, MPH; Stephen R. Benoit, MD, MPH; Susanna Naggie, MD; Joyce Frederick, MSN; Sharon Evans, RN; Jeffery Engel, MD;** and **L. Clifford McDonald, MD,** have disclosed no relevant financial relationships. **Christopher W. Woods, MD, MPH,** has disclosed the following relevant financial relationships: served as an advisor or consultant for Cepheid Diagnostics, Roche Molecular, and bioMérieux; received grants for clinical research from Cepheid Diagnostics, Roche Molecular, bioMérieux, Cubist Pharmaceuticals, and Theravance Pharmaceuticals.

## Earning CME Credit

To obtain credit, you should first read the journal article. After reading the article, you should be able to answer the following, related, multiple-choice questions. To complete the questions and earn continuing medical education (CME) credit, please go to **http://www.medscape.com/cme/eid**. Credit cannot be obtained for tests completed on paper, although you may use the worksheet below to keep a record of your answers. You must be a registered user on Medscape.com. If you are not registered on Medscape.com, please click on the New Users: Free Registration link on the left hand side of the website to register. Only one answer is correct for each question. Once you successfully answer all post-test questions you will be able to view and/or print your certificate. For questions regarding the content of this activity, contact the accredited provider, CME@medscape.net. For technical assistance, contact CME@webmd.net. American Medical Association’s Physician’s Recognition Award (AMA PRA) credits are accepted in the US as evidence of participation in CME activities. For further information on this award, please refer to http://www.ama-assn.org/ama/pub/category/2922.html. The AMA has determined that physicians not licensed in the US who participate in this CME activity are eligible for *AMA PRA Category 1 Credits*™. Through agreements that the AMA has made with agencies in some countries, AMA PRA credit is acceptable as evidence of participation in CME activities. If you are not licensed in the US and want to obtain an AMA PRA CME credit, please complete the questions online, print the certificate and present it to your national medical association.

### Article Title: Risk Factors and Estimated Incidence of Community-associated *Clostridium difficile* Infection, North Carolina, USA

## CME Questions

1. What was the approximate percentage of all *Clostridium difficile* infections (CDIs) that were community associated in the current study?
  A. 2%
  B. 7%
  C. 20%
  D. 57%
2. Which of the following demographic trends in community-associated (CA) CDI was present in the current study?
  A. Adults over the age of 64 years had the highest prevalence of CA-CDI
  B. Adults younger than 44 years had the highest prevalence of CA-CDI
  C. Physician visits had no impact on the prevalence of CA-CDI
  D. Women had a higher prevalence of CA-CDI compared with men
3. Which of the following case characteristics of CDI in the current study is most accurate?
  A. Less than 10% of patients with CDI were admitted to the hospital
  B. Diarrhea was the most common complaint
  C. Nearly all patients had been exposed to antimicrobials in the 3 months prior to testing for CDI
  D. Clindamycin was the antibiotic most frequently implicated in promoting CDI
4. All of the following variables were associated with a higher risk for CA-CDI in the current study, except:
  A. Recent treatment with antimicrobial drugs
  B. More outpatient visits at the VA hospital
  C. Treatment with proton-pump inhibitors
  D. Cardiac failure

### Activity Evaluation

**Table:** 

**1. The activity supported the learning objectives.**				
Strongly Disagree				Strongly Agree
1	2	3	4	5
**2. The material was organized clearly for learning to occur.**				
Strongly Disagree				Strongly Agree
1	2	3	4	5
**3. The content learned from this activity will impact my practice.**				
Strongly Disagree				Strongly Agree
1	2	3	4	5
**4. The activity was presented objectively and free of commercial bias.**				
Strongly Disagree				Strongly Agree
1	2	3	4	5

## Risk Factors and Estimated Incidence of Community-associated *Clostridium difficile* Infection, North Carolina, USA

*Clostridium difficile* is an anaerobic spore-forming gram-positive bacillus that produces exotoxins that are pathogenic to humans. *C. difficile* is known to infect persons receiving antimicrobial drug therapy, older and severely ill patients who are hospitalized, or residents of long-term care facilities. *C. difficile* infection (CDI) is manifested as diarrhea, pseudomembranous colitis, and, occasionally, toxic megacolon or even death. Recent reports suggest an increasing incidence and severity of CDI (*1*–*3*) that may be related to the emergence of a hypervirulent strain (*4*–*6*). In addition, reports have been published of CDI emerging in persons previously thought to be at low risk, including otherwise healthy persons in the community (*7*–*9*).

Community-associated or -acquired CDI (CA-CDI) was first reported in 1984 by Stergachis et al. (*10*) who found that the attack rate of CA antimicrobial drug–associated colitis requiring hospitalization was 1.4/100,000 population. In a review by Riley et al. (*11*) of 580 *C. difficile* toxin–positive stool samples submitted from patients with diarrhea and a clear history of recent (<4 weeks) antimicrobial drug exposure, 10.7% were from patients who did not have a recent history of hospitalization. Additional reports indicated incidences from 7.7–12.2 cases of CA-CDI per 100,000 persons in US communities (*7*,*12*) to 25 primary episodes per 100,000 persons per year in Sweden (*8*). In December 2005, the US Centers for Disease Control and Prevention (CDC) reported a series of severe CDI cases in populations previously considered at low risk, including generally healthy persons living in the Philadelphia, Pennsylvania, USA, area (*13*).

In January 2006, CDC was notified by a regional Veterans Affairs (VA) hospital in North Carolina that surveillance conducted from October 2004 through December 2005 indicated that 35% of patients with CDI experienced onset of disease in the community. Fewer than 10% of these patients had stayed overnight in a healthcare facility, and only half had received antimicrobial drugs in the previous 2 months (*14*). In response to apparent emerging CDI in the community, CDC was invited by the North Carolina Department of Health and Human Services to investigate these findings and to recommend control measures.

We retrospectively identified cases of CA-CDI in 4 North Carolina VA hospitals and all hospital catchments in Durham County, North Carolina, to determine the estimated incidence in each population. In addition, we assessed risk factors for CA *C. difficile* disease using case–control methods.

## Methods

### Settings and Case Finding

Data were collected from 6 North Carolina hospitals, including the Durham VA hospital, 2 other local hospitals, and 3 other VA hospitals located throughout the state. This activity was determined by CDC not to be human subject research; thus, human subject regulations did not apply.

Case finding involved reviewing positive *C. difficile* toxin assays from the VA infection control database, Computer Patient Record Systems, and the surveillance database of the Duke University Hospital network. Medical and laboratory records were retrospectively reviewed for case-patients from January through December 2005. Trained coders abstracted data from electronic and paper medical records using standardized forms. Data collected included demographics, signs and symptoms at the time of admission, laboratory results, prior hospitalization, risk factors, treatments, and outcomes.

### Case Definitions

A CDI case-patient was defined as a person >18 years of age with a nonformed (i.e., taking the shape of the container) stool specimen with positive test results for *C. difficile* toxin. If the case-patient had a previous *C. difficile*–positive stool test results within the 8 weeks preceding the collection date, he or she was excluded. All laboratories used the same toxin enzyme immunoassay (C. DIFFICILE TOX A/B II; TECHLAB, Blacksburg, VA, USA). CDI cases were further categorized according to when and where the stool specimen was collected, as community onset, CA, or inpatient healthcare exposure. We defined community onset as occurring 1) in an outpatient setting; 2) <3 calendar days after hospital admission; or 3) 4–5 calendar days after admission and with diarrhea documented <3 days after hospital admission. CA-CDI was defined as community-onset CDI for which there was no inpatient healthcare exposure within 8 weeks before the stool collection date. Inpatient healthcare exposure was defined as admission to an acute-care hospital or long-term care facility that provided skilled nursing care to the patient for >1 overnight stay.

We excluded CDI case-patients whose medical records appeared incomplete for determination of disease categorization; these case-patients were classified as unknowns. Community-onset CDI case-patients who were seen at Durham County hospitals but who resided outside the state were excluded because of frequent gaps in data necessary to determine the date of their last discharge from a healthcare facility and because their data could not contribute to the county population incidence. Among CA-CDI case-patients, ambulatory patients with a history of bone marrow transplant (BMT) or end-stage renal disease (ESRD) were excluded because of their intensive outpatient healthcare exposures. In addition, all prisoners were excluded. Although included in the incidence estimation, patients with inflammatory bowel disease and chronic diarrhea were excluded from the case–control study because they are recognized populations at increased risk for CDI and because their disease symptoms are difficult to differentiate from the symptoms of CDI (*15*–*17*).

We interviewed a subset of case-patients to confirm and expand information available from medical records pertaining to previous inpatient healthcare exposures and medication histories. We also surveyed physicians in Durham County to assess perceptions regarding the frequency and severity of CDI in the community and to determine laboratories used for *C. difficile* diagnostic testing. This survey included physicians working in emergency departments and in family medicine, internal medicine, obstetrics and gynecology, infectious diseases, gastroenterology, and urgent care practices. We also contacted other academic institutions and laboratories in nearby counties to determine whether our case finding was comprehensive.

#### VA Case–Control Study

We conducted an unmatched 1:3 case–control study of VA CA-CDI case-patients using controls chosen from among VA outpatients randomly selected from VA outpatient clinics seen at the 4 facilities on 4 random dates distributed throughout 2005. Exclusion criteria for controls included a documented clinical diagnosis of diarrhea or a stool test result positive for *C. difficile* toxin in 2005, a history of inpatient healthcare exposure in the prior 8 weeks, or a history of ESRD. Similar data were collected for the controls as for case-patients, except for prior hospitalization within 8 weeks.

We attempted phone interviews of all the VA CA-CDI case-patients, but not the controls, regarding their admission, symptoms, and medications. To overcome the limitation of recall bias, we reviewed electronic records after asking case-patients if they received their medications only from the VA (where electronic records were then reviewed) or whether they ever (also) obtained prescriptions from non-VA providers. All case-patient interviews were completed by the first quarter of 2006.

#### Durham County Case–Control Study

We conducted a case–control study at the 2 major hospitals serving Durham County residents. Controls were randomly selected from the county voter registration list; we made 3 attempts to reach residents by phone and elicit their participation. Exclusion criteria for controls included a history of inpatient healthcare exposure or diarrhea in the year 2005, ESRD, or BMT. We also performed phone interviews on a convenience sample of case-patients. All interviews were completed by December 2006.

### Data Analysis

Data for the case characterization and case–control study were entered into Microsoft Office Access 2003 (Microsoft Corp., Redmond, WA, USA). Data checks were performed and double entries were removed. Incidence was estimated for the North Carolina VA hospital catchments by dividing the number of CA-CDI case-patients by all veterans registered for outpatient services at the 4 VA facilities in 2005 per 100,000 person-years. An estimate of the Durham County population-based rate was determined by dividing the number of CA-CDI case-patients from Durham County (including VA case-patients who resided in the county) by the 2005 adult (>18 years of age) population census per 100,000 person-years.

Multivariable analysis was performed by using SAS version 9.1 (SAS Institute Inc., Cary, NC, USA) and stepwise logistic regression. Significant variables based on the univariate analysis (p<0.05) were included in the multivariable model. We used the Hosmer and Lemeshow and the residual χ^2^ goodness-of-fit tests. We estimated adjusted prevalence odds ratios (aORs) with 95% confidence intervals (CIs) from the regression procedure.

## Results

Of the 1,046 CDI case-patients identified from the 6 study facilities from January through December 2005, 426 (40.7%) were community-onset (Figure). A total of 214 community-onset CDI case-patients were excluded; 94 had inpatient healthcare exposures within the 8 weeks prior to positive toxin assay, 29 had ESRD, 20 were from out of state, 17 were BMT patients, 8 were prisoners, and 46 had an unknown status regarding prior inpatient healthcare exposures. The 212 case-patients with CA-CDI represented 49.5% of all cases of community-onset CDI and 20% of overall cases of CDI.

**Figure:**
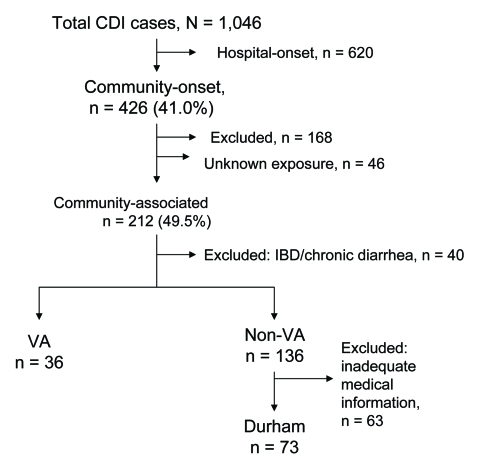
Categorization of *Clostridium difficile* infection (CDI) cases from 6 hospitals, North Carolina, 2005. IBD, irritable bowel disease; VA, Veterans Affairs hospital.

The estimated incidence of CA-CDI in the VA catchments and county adult population was 21 and 46 per 100,000 persons per year, respectively (Table 1). Among men in the VA and in Durham County, those 45–64 years of age had a higher incidence than those 18–44 and >64 years of age combined (p<0.05). Overall, incidence was higher among county female case-patients than male case-patients (62 vs. 28/100,000 persons per year, respectively; p = 0.0005).

**Table 1:** Estimated incidence of community-associated *Clostridium difficile* infection per 100,000 persons among VA and Durham County populations, North Carolina, USA, 2005*

Population	Age group, y, no. cases			Overall
	18–44	45–64	>65	
VA				
M	14.7	28.5†	15.9	20.8
Durham County				
M	11.0	56.6†	57.5	28.4
F	21.9	70.4	204.6	61.9‡
Overall	16.5	63.9	146.4	46.0

*VA, Veterans Affairs. †p<0.05 when compared with age groups 18–44 and >65 y combined. ‡p<0.005 when compared with men from Durham County.

### Characteristics of CA-CDI Cases

A total of 109 CA-CDI case-patients were found among VA catchments or Durham County residents for whom full case-characteristics were available after exclusion criteria (Figure). The characteristics of CA-CDI case-patients are described in the Appendix Table. We did not compare the 2 populations.

Nineteen case-patients in the VA catchments and 12 case-patients from Durham County had histories of hospitalization >8 weeks prior to symptom onset; medians were 33 weeks (range 10–84) and 34 weeks (range 9–92), respectively. The remaining case-patients had no recorded history of previous hospitalization. Median time from symptom onset to testing was 1 week (range 1 day–9 weeks).

Overall, 58% of case-patients in each population were admitted around the time of their CDI diagnosis, for a median duration of 1 week (maximum 3 and 5 weeks, Durham County and VA catchments, respectively). Diarrhea was the most common sign or symptom, followed by abdominal pain and vomiting. Among potential exposures, >50% of case-patients in each population had >1 outpatient visit in the 3 months before the test date. Overall, 53 case-patients (49%) did not have exposure to antimicrobial drugs in the 3 months prior to the test date. Among those who had recently received antimicrobial drug therapy, pencillins and quinolones were most commonly reported.

### Case-patient Interview

We interviewed 22 VA and 31 county (non-VA hospital) resident CA-CDI case-patients. Only 2 (4%) of the 53 patients interviewed were reclassified as other than CA-CDI on the basis of interview findings: 1 had a history of CDI 2 months before the index episode, and 1 was newly identified as an ESRD patient. Of the remaining interviewees, 25 were able to provide information on whether they had been exposed to antimicrobial drugs. Of the 11 interviewees whose medical records suggested no exposure to antimicrobial drugs within the past 3 months, 5 (45%) reported taking antimicrobial drugs during this time. These included 3 from the VA who indicated that they received all their medications from the VA; however, their electronic medical records did not have documentation of recent antimicrobial drug prescriptions.

### Case–Control Study at VA

Case-patients and controls were similar with regard to age, sex, and race (Table 2). Over the 3 months before CDI onset, exposure to antimicrobial drugs (OR 19.6, 95% CI 7.6–51, p<0.0001), antimotility medications (OR 6.6, 95% CI 1.2–37.8, p<0.05), and outpatient visits (OR 6.1, 95% CI 2–18.6, p<0.05) were most strongly associated with case status. Among antimicrobial drugs, exposure to penicillins and quinolones were significant. In the multivariable analysis, antimicrobial drug use with an aOR of 17.8 (95% CI 6.6–48, p<0.0001) and outpatient visits (aOR 5.1, 95% CI 1.5–17.9, p<0.05) were significant risk factors.

**Table 2:** Results of community-associated *Clostridium difficile* infection case–control study, Veterans Affairs, North Carolina, USA, 2005*

Characteristic	No. (%) persons		OR (95% CI)
	Case-patients, n = 36	Controls, n = 108	
Demographics			
Median age, y (range)	62 (38–85)	64 (36–86)	
Female gender	4 (11)	4 (4)	
Non-Hispanic white race	25 (69)	70 (65)	
Coexisting health conditions			
Hypertension	16 (44)	75 (69)	0.35 (0.2–0.8)†
Cardiac failure	6 (17)	5 (5)	4.1 (1.2–14.4)†
Exposures‡			
Outpatient visit	32 (89)	69 (57)	6.1 (2.0–18.6)†
Antimicrobial drugs	24 (66)	10 (9)	19.6 (7.6–51.0)†
Penicillins	13 (36)	3 (3)	19.8 (5.2–75.1)§
Quinolones	6 (17)	3 (3)	7 (1.7–29.7)†
NSAIDs	6 (17)	30 (28)	0.5 (0.2–0.4)
Antimotility medications	4 (11)	2 (2)	6.6 (1.2–37.8)†
Gastric acid suppressors	18 (50)	37 (34)	1.9 (0.9–4.1)
H2 blockers	7 (19)	13 (12)	1.8 (0.6–4.8)
Proton pump inhibitors	13 (36)	26 (24)	1.7 (0.7–4.0)
Steroids	4 (11)	3 (3)	4.4 (0.9–20.5)
Multivariable analysis¶			
Antimicrobial drugs			17.8 (6.6–48.0)#
Outpatient visit			5.1 (1.5–17.9)†#

*OR, odds ratio; CI, confidence interval; NSAIDs, nonsteroidal antiinflammatory drugs. †p<0.05. ‡Exposures among case-patients and controls were within 3 months prior to the test date. §p< 0.0001. ¶Goodness-of-fit tests: residual χ^2^, p = 0.33; Hosmer and Lemeshow, p = 0.12. #Adjusted OR.

### Case–Control Study in Durham County

Information was obtained for all 73 case-patients but only for 48 controls (Table 3). The case-patients and controls were similar with regard to age, sex, and race. Among potential exposures during the previous 3 months, exposure to antimicrobial drugs (OR 3.9, 95% CI 1.6–0.5, p<0.05) was most strongly associated with case status. In the multivariable analysis, antimicrobial drug use (aOR 9.1, 95% CI 2.9–28.9, p<0.05), gastroesophageal reflux disease (GERD) (aOR 11.2, 95% CI 1.9–64.2, p<0.05), and cardiac failure (aOR 3.8, 95% CI 1.1–13.7, p<0.05) were independent risk factors.

**Table 3:** Results of community-associated *Clostridium difficile* infection case–control study, Durham County, North Carolina, USA, 2005*

Characteristics	No. (%) persons		OR (95% CI)
	Case-patients, n = 73	Controls, n = 48	
Demographics			
Median age, y (range)	61 (20–101)	55 (22–87)	
Female gender	57 (58)	34 (71)	
Non-Hispanic white race	34 (47)	35 (73)	0.32 (0.12–0.7)†
Coexisting health conditions			
Cardiac failure	20 (27)	5 (10)	3.24 (1.12–9.4)†
GERD	20 (27)	2 (4)	8.7 (1.9–39.1)†
Hypertension	36 (49)	13 (27)	2.6 (1.2–5.7)†
Exposures‡			
Antimicrobial drugs	32 (44)	8 (17)	3.9 (1.6–9.5)†
NSAIDs	13 (17.8)	24 (50)	0.2 (0.1–0.5)†
Gastric acid suppressors	15 (21)	5 (10)	2.2 (0.75–6.6)
H2 blockers	6 (8)	3 (6)	1.3 (0.3–5.6)
Proton pump inhibitor	9 (12)	2 (4)	3.2 (0.7–15.7)
GERD	20 (27)	2 (4)	8.7 (1.9–39.1)†
Multivariable analysis§			
Antimicrobial drugs			9.1 (2.9–28.9)†¶
NSAIDs			0.2 (0.1–0.5)†¶
GERD			11.2 (1.9–64.2)†¶
White race			0.2 (0.05–0.40)†¶
Cardiac failure			3.8 (1.1–13.7)†¶

*OR, odds ratio; CI, confidence interval; GERD, gastroesophageal reflux disease; NSAIDs, nonsteroidal antiinflammatory drugs. †p<0.05. ‡Exposures among case-patients were within 3 months prior to the test date; exposures for controls were for all of 2005. §Goodness-of-fit tests: residual χ^2^, p = 0.38; Hosmer and Lemeshow, p = 0.77. ¶Adjusted OR.

### Physician Survey

Of 33 Durham County primary care and relevant specialty medicine practices surveyed, 59 physicians from 16 (48.5%) practices responded. Only 2 commercial laboratories, in addition to the 2 Durham hospital laboratories, were used for diagnostic testing of *C. difficile* in the outpatient setting. One laboratory was unable to provide any information regarding positive *C. difficil*e tests performed on persons living in Durham County, whereas the other laboratory had 14 positive *C. difficile* test results for this population during 2005. Additional information about these cases was unavailable.

## Discussion

We found that 20% of all CDI cases were CA, similar to the 22%–28% found in previous studies conducted in Sweden (*8*,*18*). We are not aware of previous studies from the United States that determined the proportion of CA-CDI from all CDI cases. However, available literature on the incidence of CA-CDI in the United States (*7*,*13*,*19*) reports lower rates than what we found in the VA (21/100,000 persons/year) and Durham County (46/100,000 persons/year) catchments. The annual incidence in Philadelphia in 2005 (7.6/100,000 persons/year) and the incidence in Connecticut in 2006 (6.9/100,000 persons/year) were based on passive, voluntary reports of CA-CDI in mostly healthy persons and thus likely underestimated the true incidence and spectrum of disease (*13*,*19*). However, a recent active case-finding study for CA-CDI conducted in the United Kingdom (*20*) suggested rates of 20–30 per 100,000, similar to the studies conducted in Sweden (*8*,*18*). Furthermore, a recent study from Manitoba, Canada, found that ≈20% of CDI cases were CA, and annual incidence was ≈20/100,000 (*21*). Apparent differences in the incidence of CA-CDI likely reflect differences both in study approaches and population characteristics. Nonetheless, CA-CDI should be considered a serious public health concern in need of further understanding and improved surveillance. Although it is unclear why the estimated incidence rates in Durham County are the highest for CA-CDI reported, recent evidence indicate that the rates in the county may have since declined (*22*), a phenomenon anecdotally experienced in other locations in the southeastern United States (R.P. Gaynes, pers. comm.) (*23*).

The incidence rate we found was highest for the 45–64 years of age category (28.5/100,000) for the VA catchments. In Durham County, men 45–64 years of age had a higher rate than other those in age categories combined (p<0.05). This finding is notable because CDI is usually associated with older patients (*9*,*19*). Women had a higher rate than males in the community (62 vs. 28 cases/100,000 persons/year, respectively; p = 0.0005), as was also recently noted in a report from Connecticut (*19*). Although some data suggest an increased risk for multiple CDI recurrences in healthy women (*24*) and recent reports have noted severe CDI in pregnant women (*25*), female gender has not been previously a well-documented risk factor for CDI.

Previous reports have shown that an age >65 years is a risk factor for hospital-onset CDI (*2*,*26*,*27*). In comparison, the median age of the case-patients in this community appeared to be younger, 61 years in Durham County and 63 years in VA. In addition, the proportion and severity of fever, leukocytosis, and renal insufficiency for our case-patients were lower (*26*,*28*,*29*). No case-patients were admitted to the intensive care unit for CA-CDI, and none underwent colectomy. One case-patient in each population died within 10 days of diagnosis, and the death was attributable to CDI. Nonetheless, 15% had disease severe enough to require a visit to the emergency department, and another 59% required hospital admission for CDI management.

Antimicrobial drug exposure has long been known as a risk factor for healthcare-associated CDI. However, among CA cases in our study, 49% were not exposed to antimicrobial drugs. This percentage was slightly higher than the recent findings from Philadelphia (*13*) and Connecticut (*19*), where 24% and 36%, respectively, were not exposed to antimicrobial drugs. In contrast, Dial et al. found the absence of antimicrobial drug exposure >90 days to range from 60% to 70% (*30*). However, their analysis was limited to a clinical research database in which some hospitalization and antimicrobial drug exposures may not have been included. Two prospective studies have recently been conducted in the community. Bauer et al. (*31*) found that 42% of CA-CDI case-patients had not been exposed to antimicrobial drugs during the prior 6 months, and Wilcox et al. (*20*) found that 84% case-patients had not received antimicrobial drugs during the month before *C. difficile* detection. One hypothesis to explain the absence of antimicrobial drug exposure is that there are unmeasured factors affecting the epidemiology of CDI. For example, remote antimicrobial drug exposure, or exposure to other medications with antimicrobial activity, may be increasing the risk of disease; alternatively, increased awareness of CDI may be leading to increased testing and documentation of *C. difficile* in patients not previously tested. Another possibility is that strains with new virulence properties (e.g., binary toxin) that enable disease in the absence of prior antimicrobial drug use have emerged. Despite antimicrobial drug exposure being absent in many patients, we found that this exposure remained the most important modifiable risk factor for CA-CDI.

Additional risk factors included markers of chronic disease such as outpatient visits in the VA population and GERD and cardiac failure in the county population. In the VA population, frequent outpatient visits could reflect transmission in ambulatory care settings and could be a marker of a more severe underlying disease. Unlike other recent studies (*9*), we did not find proton pump inhibitors or H2 blockers were a major risk factor for CA-CDI. However, the finding of GERD as a risk factor suggests the possibility that undocumented use of over-the-counter proton pump inhibitors could have increased risk in these patients. Alternatively, there may be factors in the pathogenesis of GERD that increases the risk for CDI.

Our study has several limitations. First, it is likely that there was incomplete case ascertainment, especially in those who underwent testing by outside laboratories so that, as high as these population incidence estimates are, they are likely underestimates of the true incidence. However the degree of underestimate is less likely in the VA system as there is financial incentive for patients to undergo testing within the system. The community physician survey conducted in Durham County indicated that there were 2 commercial laboratories other than Durham hospital laboratories used for testing. Although we were unable to determine the number of *C. difficile* tests performed at 1 laboratory, only 14 case-patients were identified from the other. It is also possible that patients received empiric therapy for CDI without a test being performed. However, the same survey of Durham County physicians indicated this was not a common practice. Some potential case-patients were categorized as unknowns when little or no medical records were available. We did not collect data on laboratory testing performed for any other enteric pathogens besides *C. difficile* nor did we perform cultures for *C. difficile,* and therefore no isolates were available.

Instead, case confirmation was limited to toxin immunoassay testing using the C. DIFFICILE TOX A/B II TECHLAB test. In an independent review (*32*), the sensitivity of this test was 83.3% and the specificity was 98.7%. To address the concern of inadequate sensitivity in the toxin immunoassay and to avoid any misclassification bias in our case–control studies, we excluded controls who had diarrhea. Despite the high specificity of this test, there are valid concerns that if a low-prevalence population, such as relatively asymptomatic persons without prior antimicrobial drug exposures, is tested, the likelihood of a false-positive result may be unacceptably high. Although this is an insurmountable obstacle to a retrospective analysis of current clinical testing practice for CDI, the fact that all study laboratories had rejection criteria to prevent testing formed stool, near uniform medical record documentation of diarrhea (i.e., patients with documented absence of diarrhea were excluded), and a median duration of diarrhea symptoms of 1 week suggests a reasonable pretest likelihood of CDI among these patients.

Another limitation was that few interviews were performed with case-patients. However, the adequacy of records indicating exposure to inpatient healthcare or antimicrobial drugs was verified among 53 case-patients who were interviewed by telephone. Only 1 case-patient was reclassified on the basis of an undocumented healthcare exposure, which was discovered during the interview process. Five of the 11 case-patients for whom antimicrobial drug exposure was not identified in their available medical records reported antimicrobial drug use. However, 3 of these were VA case-patients for whom medical records did not document such use, which suggests that some patients may have been mistaken about their antimicrobial drug exposure.

Another limitation is that our use of outpatient controls for the VA case–control study may have resulted in bias toward the null with regard to outpatient healthcare-related risk factors. Although we attempted to contact >400 candidate controls from the voter registration list for the Durham County case–control study, we encountered difficulty in reaching persons by phone and eliciting their participation. This resulted in only 48 controls being available and limited the power of this analysis.

In summary, CA-CDI is a relatively common clinical diagnosis. Although we did not determine the incidence in children, we found that CA-CDI in Durham County has a spectrum of disease that involves predominantly middle and older-aged women with underlying illness. As previously documented in other recent studies, this disease may, and commonly does, occur in patients without recent antimicrobial drug exposure. Nonetheless, antimicrobial drug exposure use remains the most important modifiable risk factor, suggesting prudent antimicrobial use remains a prominent public health prevention strategy. Further research into the incidence, sources, and risk factors for CA-CDI should be an ongoing public health priority.

## Supplementary Material

Appendix Table Characteristics of case-patieints with community-associated Clostridium difficile infection, North Carolina, USA, 2005
