# Supplementary material for: Risk Factors for and Estimated Incidence of Community-associated Clostridium difficile Infection, North Carolina, USA1
Source: Emerg Infect Dis. 2010 Feb;16(2):198–204. doi: 10.3201/eid1602.090953 (PMC2958012; doi:10.3201/eid1602.090953)
Supplement: Appendix Table — Characteristics of case-patieints with community-associated Clostridium difficile infection, North Carolina, USA, 2005 [file 09-0953_appT-s1.pdf]

Online Appendix Table. Characteristics of case-patieints with community-associated *Clostridium difficile* infection, North Carolina, USA, 2005

Characteristics	No. (%) case-patients	
	Durham County, n = 73	Veterans Affairs, n = 36
Demographics		
Median age, y (range)	61 (20–101)	63 (38–85)
Female gender	57 (78)	4 (11)
Race*		
Non-Hispanic white	34 (47)	25 (70)
Non-Hispanic black	24 (33)	8 (22)
Other	15 (20)	3 (8)
Coexisting health conditions		
Hypertension	36 (49)	16 (44)
Cardiovascular disease	20 (27)	14 (39)
Gastroesophageal reflux disease	20 (27)	11 (31)
Diabetes	17 (23)	13 (36)
Bowel surgery	5 (7)	2 (6)
Others†	20 (27)	27 (75)
Exposures within 3 months before test date		
Outpatient visit	37 (51)	32 (89)
Antimicrobial drugs	32 (44)	24 (67)
Penicillins	7 (22)	13 (54)
Quinolones	9 (28)	6 (25)
Lincosamides (clindamycin)	8 (11)	2 (6)
Cephalosporins	3 (9)	2 (8)
Macrolides	2 (6)	2 (8)
Proton pump inhibitors	9 (12)	13 (36)
NSAIDS‡	13 (18)	6 (16)
Antimotility drugs	8 (11)	4 (11)
H2 blockers	6 (8)	7 (19)
Laxatives	3 (4)	7 (19)
Steroids	4 (6)	4 (11)
Patient location at the time of testing		

Outpatient	46 (63)	16 (44)
Inpatient	27 (37)	20 (56)
Documented signs and symptoms		
Diarrhea§	62 (85)	33 (92)
Bloody diarrhea	15 (24)	6 (18)
Abdominal pain	43 (59)	21 (58)
Vomiting	20 (27)	16 (44)
Fever on the date of visit (>100.4°F oral)	9 (12)	5 (14)
Laboratory		
Leukocytes/mL within 1 week	n = 49	n = 25
Median	11,353	10,144
Range	300–33,400	2,400–22,000
Serum creatinine, mg/dL (highest level during admission)	n = 54	n = 30
Median	1	1.2
Range	0.5–4	0.7–3.3
Treatment given		
Metronidazole	53	31
Vancomycin	3	4
Unknown	19	5
Outcome		
Visit to outpatient physician	16 (22)	9 (25)
Visit to emergency room but no admission	11 (15)	5 (14)
Admission to hospital	42 (58)	22 (61)
Death due to *C. difficile* infection	1	1
Unknown	4 (5)	0

*Race was defined as per the documentation in the electronic and paper medical records.

†Others include diverticular disease, cancer, chronic obstructive pulmonary disease, gastric surgery, HIV infection, cirrhosis, anemia, organ transplant, methicillin-resistant *Staphylococcus aureus* infection.

‡Nonsteroidal antiinflammatory drugs.

§Diarrhea as documented in available medical records; all patients had an unformed stool submitted to the laboratory.

[1]Presented in part at the 44th Annual Meeting of the Infectious Diseases Society of America, Toronto, Ontario, Canada, October 12–15, 2006.
